# DNA methylation inhibitors adverse reaction characteristic analysis: a descriptive analysis from WHO-VigiAccess

**DOI:** 10.3389/fphar.2024.1470148

**Published:** 2024-10-02

**Authors:** Qiang Zhou, Quanlei Xie, Qiang Liu, Haojie Wang, Zhan Zhang, Zhao Yu, Qian Guo, Jie Lin

**Affiliations:** ^1^ Department of Pharmacy, The Third Affiliated Hospital of Wenzhou Medical University (Ruian People’s Hospital), Wenzhou, China; ^2^ Department of Vascular Surgery, The First Affiliated Hospital of Nanchang University, Nanchang, China; ^3^ Department of Neurosurgery, The First Affiliated Hospital of Harbin Medical University, Harbin, China; ^4^ Department of Rhinology, The First Affiliated Hospital of Zhengzhou University, Zhengzhou, China

**Keywords:** DNA methylation inhibitors, myelodysplastic syndromes, WHO-vigiaccess, retrospective descriptive analysis, adverse reaction

## Abstract

**Introduction:**

DNA methylation inhibitors (azacitidine, decitabine) have revolutionized the treatment dilemma of myelodysplastic syndromes (MDS), a group of malignant hematopoietic disorders. This study evaluates the adverse drug reactions (ADRs) following the use of DNA methylation inhibitors in the World Health Organization (WHO) VigiAccess database and compares the characteristics of ADRs between the two drugs to select the drug with the minimum individualized risk for patients.

**Methods:**

This study employed a retrospective descriptive analysis method. We compiled ADR reports for two marketed DNA methylation inhibitors for the treatment of MDS from WHO-VigiAccess. Data collected included demographic data such as age groups, gender, and regions of global patients covered by ADR reports, as well as data on the disease systems and symptoms caused by ADRs recorded in the annual reports and reports received by WHO. By calculating the proportion of ADRs reported for each drug, we compared the similarities and differences in ADRs between the two drugs.

**Results:**

Overall, 23,763 adverse events (AEs) related to the two DNA methylation inhibitors were reported in VigiAccess. The results showed that the top 10 most common AEs were febrile neutropenia, bone marrow suppression, neutropenia, anemia, pancytopenia, leukopenia, thrombocytopenia, bone marrow failure, agranulocytosis, and hematotoxicity. The top five common types of DNA methylation inhibitor AEs were blood and lymphatic system disorders (11,178 cases, 47.0%), cardiac organ diseases (1,488 cases, 6.3%), various congenital familial genetic diseases (49 cases, 0.2%), ear and labyrinth diseases (100, 4.2%), and endocrine system diseases (57, 2.4%).

**Conclusion:**

There is no Strong correlation between DNA methylation inhibitors and ADRs. Current comparative observational studies of these inhibitors show that there are common and specific adverse reactions in the ADR reports received by WHO for these drugs. Clinicians should improve the rational use of these drugs based on the characteristics of ADRs.

## Introduction

Myelodysplastic Syndromes (MDS) are a group of heterogeneous chronic hematologic malignancies characterized by impaired bone marrow hematopoiesis and ineffective hematopoiesis, as well as a variable risk of progression to acute myeloid leukemia (AML). MDS is driven by a complex combination of genetic mutations, leading to heterogeneous clinical phenotypes and outcomes. Genetic studies have been able to identify a set of genes with recurrent mutations that are central to the pathogenesis of MDS (Chiereghin et al., 2021). DNA methylation is essential for imprinting, X inactivation, and the silencing of pluripotent or tissue-specific genes, thereby regulating embryonic development. It is also necessary to maintain chromosomal stability in differentiated cells and to prevent mutations by inhibiting the insertion of transposons and repetitive elements. Therefore, the failure to maintain these epigenetic marks and the establishment of abnormal DNA methylation patterns are associated with the underexpression or overexpression of certain proteins, ultimately leading to various pathologies (Gros et al., 2012). Thus, DNA methylation inhibitors can effectively treat MDS. At present in the clinic, azacitidine (AZA) and decitabine (DAC) are the most widely used methylation inhibitors (Sekeres and Taylor, 2022). Studies have shown that azacitidine and decitabine play a very important role in the treatment of chronic hematologic malignancies such as MDS. Regarding its mechanism of action, there are many hypotheses in academia, among which the view that “the activity of DNA methyltransferase is inhibited, leading to hypomethylation of tumor suppressor genes and upregulation of tumor suppressor gene expression” is widely recognized. In fact, DNA methylation inhibitors often act at the whole genome level, and their global impact not only includes causing demethylation of tumor suppressor genes and upregulating the expression of tumor suppressor genes, thereby exerting therapeutic effects, but may also include inducing demethylation of oncogenes, thereby leading to the upregulation of oncogenes and producing pathogenic effects. Therefore, in the treatment of MDS, the potential “innate insufficiency” of DNA methylation inhibitor treatment is that while demethylating tumor suppressor genes, it also upregulates the expression of oncogenes, not only treating the disease but also carrying a very high risk of pathogenicity (Liu et al., 2022). According to existing data, the efficacy of DNA methylation inhibitors in patients with myelodysplastic syndrome and acute myeloid leukemia is also far lower than expected in the clinic, some patients do not respond to this type of drug, and a few patients have an average survival period of less than half a year after the failure of DNA methylation inhibitor treatment, and the upregulation of oncogenes may be an important reason. This indicates that the applicable population of demethylation therapy is limited, and the clinic needs to carry out more targeted group treatments. More importantly, although both have been approved for clinical treatment, there is currently less research comparing the similarities and differences in adverse reactions caused by the two.

This study retrieved two demethylation drugs for the treatment of MDS approved by the US Food and Drug Administration (FDA): azacitidine and decitabine. These two therapeutic drugs showed similar efficacy characteristics. As of 31 July 2020, according to a meta-analysis using markov chain monte carlo method to network meta-analysis, the primary end point for overall survival (OS) and the incidence of adverse events, and secondary endpoints were complete response rate (CR), the total response rate (ORR) and no AML surial. There are six randomized controlled trials involving 1,072 patients with MDS, three randomized controlled trials, involving 1,256 patients with AML. The meta-analysis showed that in MDS, AZA showed a better AML-free survival period (risk ratio = 0.62; 95% CI, 0.43–0.9), while DAC may achieve better CR and ORR, and AZA may obtain better OS and lower toxicity. For elderly AML patients, DAC may achieve better CR, ORR, and OS, but the toxicity is relatively higher. In addition, subgroup analysis of patients aged 75 or MDS high-risk patients ≥ showed that AZA achieved better OS (Liu et al., 2021). Therefore, clinicians usually need to tailor treatment decisions according to the risk of adverse events for individual patients, and we conducted a descriptive study of spontaneously reported adverse reactions in the VigiAccess database to compare the adverse reaction reporting rates caused by the two drugs.

## Materials and methods

### Drug samples

Table 1 shows the two demethylation drugs for the treatment of MDS that we have studied for clinical research.

**TABLE 1:** Overview of two DNA methylation inhibitors.

Drug name	Chemical name	Structure	Main treatment	First marketed year
Azacitidine	4-amino-1-β-D-ribofuranosyl-1,3,5-triazine-2(1H)-one	C8H12N4O5	Myelodysplastic syndromes (MDS), Acute Myeloid Leukemia (AML), and Chronic Myeloid Leukemia	2004
Decitabine	5-azacitidine-2′-deoxycytidine	C8H12N4O4	Myelodysplastic Syndromes (MDS) and Acute Myeloid Leukemia (AML)	2012

Azacitidine and decitabine are both drugs used to treat certain types of cancer, and they have different chemical structures and mechanisms of action. Azacitidine (Azacitidine for Injection) is the only DNA methylation inhibitor that can significantly extend the overall survival of high-risk MDS patients, the first MDS treatment drug approved by the US FDA, and recommended by the US National Comprehensive Cancer Network guidelines as a first-line treatment drug. This product is suitable for the treatment of all subtypes of MDS and has the qualification of rare disease treatment drugs. It is a cytidine nucleoside analog that exerts anti-tumor effects by causing DNA demethylation and direct cytotoxic effects on abnormal hematopoietic cells in the bone marrow, mainly used for the treatment of myelodysplastic syndrome (MDS), chronic myelomonocytic leukemia (CMML), and acute myeloid leukemia (AML). It also has certain efficacy against breast cancer, colon cancer, melanoma, *etc.* Decitabine is an adenosine analog of natural 2′-deoxycytidine acid, which inhibits DNA methyltransferase, reduces DNA methylation, thereby inhibiting tumor cell proliferation and preventing drug resistance. Decitabine inhibits DNA methylation *in vitro* but does not affect DNA synthesis, mainly used for the treatment of myelodysplastic syndrome (MDS), and has anti-tumor activity, showing a dual mechanism of dose difference: cytotoxicity at high concentrations and demethylation at low concentrations (Lee et al., 2013).

### Data source

WHO-VigiAccess was searched on 17 July 2024, to find all adverse events reported after the introduction of demethylation therapy drugs for MDS. The login website is https://www.vigiaccess.org. All research drugs were identified by their generic names. WHO-VigiAccess collects data on age groups, gender, reporting year, and continents around the world. Descriptive data were calculated using Excel 2016. WHO-VigiAccess is a free portal of the PIDM database, allowing the retrieval of drug safety reports received by UMC. This definition depends on the System Organ Class (SOC) and Preferred Term (PTs) of the Medical Dictionary for Regulatory Activities (MedDRA). Therefore, records of each drug were retrieved, and all individual AEs were determined according to the SOC and PT levels recorded to describe the toxicity spectrum. The reporting terms used in MedDRA come from several dictionaries, including the World Health Organization Adverse Reaction Terminology (WHO-art), *etc.* (Sultana et al., 2020). The SOC classification has a total of 27 entries, and 20 entries directly related to disease symptoms were selected for analysis. In this study, we focused on the PTs, that is, the levels used in the VigiBase database publicly accessed through WHO-VigiAccess. To study the results of the detected safety signals, we grouped them using the outcome codes to produce three serious categories: death, hospitalization, and major events including life-threatening events, disabilities, and congenital abnormalities.

### Statistical analysis

This study used a retrospective quantitative research design. Descriptive analysis using Excel was performed to analyze the characteristics of the victims of adverse reactions to the two drugs. The number of ADR symptoms for each drug divided by the total number of ADR reports is defined as the ADR reporting rate for that drug. The common ADRs for each drug refer to the ADR reporting rate of the top 20 symptoms. The incidence of ADR symptoms reported for each drug was calculated and a descriptive comparative analysis was performed. Descriptive variables were categorized using frequency and percentage.

## Results

Study Medical Record Description: The earliest adverse reaction reports for azacitidine and decitabine received in the WHO-VigiAccess database were in 1978 and 2003, respectively. As of 2024, the World Health Organization has received a total of 17,925 and 5,838 adverse reaction reports for these two drugs, totaling 23,763. The number of adverse events covered in these adverse reaction reports is 42,335 for azacitidine and 14,390 for decitabine. In the 23,763 reports related to the two DNA methylation inhibitor drugs shown in Table 2.

**TABLE 2:** Characteristics of ADR reports of Two DNA Methylation Inhibitors.

	Azacitidine	Decitabine
Number of ADR reports	17,925	5,838
Female	6,298 (35.1%)	2022 (34.6%)
Male	9,905 (55.3%)	3,186 (54.6%)
Unknown	1722 (9.6%)	630 (10.8%)
<18	240 (1.3%)	121 (2.1%)
18–44	734 (4.1%)	441 (7.6%)
45–64	3,290 (18.4%)	1,333 (22.8%)
65–74	5,221 (29.1%)	1728 (29.6%
>75	4,745 (26.5%)	1,331 (22.8%)
Unknown	3,695 (20.6%)	884 (15.1%)
Africa	43 (0.2%)	3 (0.1%)
Americas	5,860 (32.7%)	1741 (29.8%)
Asia	5,594 (31.2%)	3,528 (60.4%)
Europe	5,805 (32.4%)	556 (9.5%)
Oceania	623 (3.5%)	10 (0.2%)
Before 2010	845 (4.7%)	389 (6.7%)
2011	598 (3.3%)	137 (2.3%)
2012	606 (3.4%)	77 (1.3%)
2013	1,131 (6.3%)	92 (1.6%)
2014	1,294 (7.2%)	333 (5.7%)
2015	1,374 (7.7%)	545 (9.3%)
2016	1,332 (7.4%)	585 (10%)
2017	945 (5.3%)	437 (7.5%)
2018	1,076 (6%)	307 (5.3%)
2019	1,212 (6.8%)	444 (7.6%)
2020	1,053 (5.9%)	712 (12.2%)
2021	1,496 (8.3%)	444 (7.6%)
2022	1892 (10.6%)	383 (6.6%)
2023	1993 (11.1%)	560 (9.6%)
2024	1,078 (6%)	393 (6.7%)

Except for 2,332 cases with unknown gender, the number of men experiencing adverse reactions (13,191) is significantly more than that of women (8,320), with a male-to-female ratio of 1.59:1, a significant difference. Excluding reports with unknown age, the age group with the highest reporting rate is mostly between 65 and 74 years old. Most of the reported AEs come from Asia (38.38%). Table 2 also lists the reporting years for each study drug.

### Distribution of 20 System Organ Classes (SOCs) for two DNA methylation inhibitors

Table 3 and Supplementary Table S1 show the reporting rates of the 20 SOCs for the two DNA methylation inhibitors. Azacitidine-related hematologic and lymphatic system disorders, cardiac disorders, gastrointestinal disorders, nervous system disorders, respiratory, thoracic and mediastinal disorders, and vascular disorders have significantly higher reporting rates than decitabine. In addition, general disorders and administration site conditions, infections and parasitic infestations, the number of examinations, benign, malignant, and unspecified tumors, including cysts and polyps, are also significantly more numerous for azacitidine than for decitabine. The top five most commonly reported AE types for DNA methylation inhibitors are: blood and lymphatic system disorders (8,968 cases, 37.74%), general disorders and administration site conditions (5,784 cases, 24.34%), infections and parasitic infestations (5,379 cases, 22.63%), gastrointestinal disorders (3,562 cases, 14.99%), and examinations (3,363 cases, 14.15%).

**TABLE 3:** ADR number and report rate of 20 SOCs of Two DNA Methylation Inhibitors.

System organ class	Azacitidine (N = 17,925)	Decitabine (N = 5,838)
Blood and lymphatic system disorders	6,403 (35.72%)	2,565 (43.94%)
Cardiac disorders	974 (5.43%)	234 (4.01%)
Congenital familial and genetic disorders	59 (0.33%)	17 (0.29%)
Ear and labyrinth disorders	66 (0.37%)	28 (0.48%)
Endocrine disorders	36 (0.2%)	5 (0.09%)
Eye disorders	113 (0.63%)	60 (1.03%)
Gastrointestinal disorders	2,568 (14.33%)	994 (17.03%)
General disorders and administration site conditions	4,457 (24.86%)	1,327 (22.73%)
Hepatobiliary disorders	354 (1.97%)	100 (1.71%)
Immune system disorders	273 (1.52%)	75 (1.28%)
Infections and infestations	4,213 (23.5%)	1,166 (19.97%)
Injury poisoning and procedural complications	1,117 (6.23%)	487 (8.34%)
Investigations	2,585 (14.42%)	778 (13.33%)
Metabolism and nutrition disorders	862 (4.81%)	444 (7.61%)
Musculoskeletal and connective tissue disorders	530 (2.96%)	243 (4.16%)
Neoplasms benign malignant and unspecified incl cysts and polyps	1836 (10.24%)	373 (6.39%)
Nervous system disorders	1,037 (5.79%)	392 (6.71%)
Pregnancy puerperium and perinatal conditions	5 (0.03%)	2 (0.03%)
Product issues	74 (0.41%)	3 (0.05%)
Psychiatric disorders	287 (1.6%)	121 (2.07%)
Renal and urinary disorders	682 (3.8%)	207 (3.55%)
Reproductive system and breast disorders	54 (0.3%)	22 (0.38%)
Respiratory thoracic and mediastinal disorders	1,602 (8.94%)	558 (9.56%)
Skin and subcutaneous tissue disorders	1,317 (7.35%)	330 (5.65%)
Social circumstances	68 (0.38%)	14 (0.24%)
Surgical and medical procedures	104 (0.58%)	38 (0.65%)
Vascular disorders	628 (3.5%)	194 (3.32%)

### The most common adverse reactions for two DNA methylation inhibitors

Table 4 lists the 20 most commonly reported adverse reactions for the two inhibitors, presented as preferred terms within the SOC. The common adverse reactions for all DNA methylation inhibitors are febrile neutropenia, neutropenia, bone marrow suppression, thrombocytopenia, anemia, pancytopenia, leukopenia, cytopenia, bone marrow failure, hematotoxicity, agranulocytosis, disseminated intravascular coagulation, granulocytopenia, platelet disorders, febrile bone marrow hypoplasia, splenomegaly, leukocyte disorders, thrombocythemia, and hemolysis. Compared with azacitidine, decitabine has a significantly higher reporting rate for adverse reactions related to immune responses.

**TABLE 4:** Top 20 ADRs of two DNA methylation inhibitors.

Azacitidine (N = 17,925)		Decitabine (N = 5,838)	
ADR	Report rate (%)	ADR	Report rate (%)
Febrile neutropenia	10.18	Myelosuppression	11.66
Neutropenia	6.67	Neutropenia	9.30
Myelosuppression	6.14	Febrile neutropenia	8.12
Thrombocytopenia	6.03	Thrombocytopenia	7.66
Anaemia	4.65	Leukopenia	5.24
Pancytopenia	3.04	Anaemia	3.82
Leukopenia	2.05	Pancytopenia	2.95
Cytopenia	1.60	Bone marrow failure	0.65
Bone marrow failure	0.59	Cytopenia	0.58
Haematotoxicity	0.57	Agranulocytosis	0.53
Agranulocytosis	0.51	Granulocytopenia	0.34
Leukocytosis	0.36	Haematotoxicity	0.31
Disseminated intravascular coagulation	0.26	Leukocytosis	0.27
Granulocytopenia	0.21	Thrombocytosis	0.17
Platelet disorder	0.19	Bicytopenia	0.15
Febrile bone marrow aplasia	0.18	Splenomegaly	0.14
Splenomegaly	0.16	Erythropenia	0.10
White blood cell disorder	0.13	Coagulopathy	0.09
Thrombocytosis	0.12	Disseminated intravascular coagulation	0.09
Haemolysis	0.11	Haemolytic anaemia	0.09

### 2 types of DNA methylation inhibitors have serious adverse events

**FIGURE 1:**
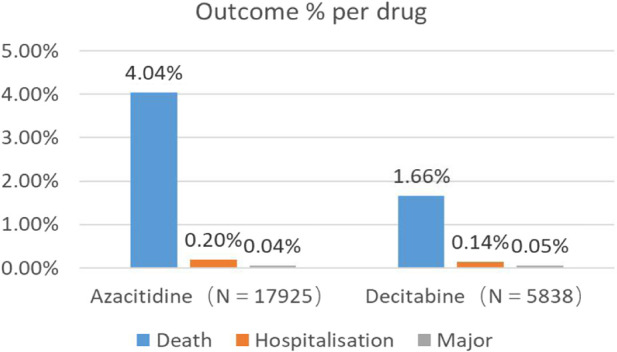
Outcomes for serious adverse events associated with DNA Methylation Inhibitors at the level of preferred terms (major events comprising life-threatening events, disability, and congenital anomaly).

Through WHO-VigiAccess, we can also find that the main adverse events of DNA methylation inhibitors include death, hospitalization, and life-threatening events. The proportion of deaths caused by Azacitidine and Decitabine are 4.04% and 1.66% respectively (Figure 1).

### Same and different adverse reactions of two DNA methylation inhibitors

By comparing the top 27 adverse reactions reported for each DNA methylation inhibitor in the System Organ Class (SOC), 173 common signals were found at the Preferred Term (PT) level for the two inhibitors. All common signals are categorized in Table 5. The SOC with the most adverse reaction signals is General Disorders and Administration Site Conditions, with the top five reported being pain, drug ineffectiveness, multiple organ dysfunction syndrome, chest pain, and disease progression. Additionally, for Respiratory, Thoracic and Mediastinal Disorders, the top five reports are cough, interstitial lung disease, pneumonia, pulmonary infiltration, and acute respiratory distress syndrome.

**TABLE 5:** Same ADRs among two DNA methylation inhibitors.

System organ classes	ADRs	Signal N
Blood and lymphatic system disorders	Neutropenia, Cytopenia, Anaemia, Bone marrow failure, Agranulocytosis, Splenomegaly, Thrombocytosis, Myelosuppression, Thrombocytopenia, Pancytopenia, Haematotoxicity, Granulocytopenia, Febrile neutropenia, Leukopenia, Leukocytosis	15
Cardiac disorders	Cardiac failure congestive, Cardiac arrest, Acute myocardial infarction, Atrial fibrillation, Pericardial effusion, Tachycardia, Supraventricular tachycardia, Cardiac failure, Myocardial infarction	9
Congenital, familial and genetic disorders		
Ear and labyrinth disorders		
Endocrine disorders		
Eye disorders		
Gastrointestinal disorders	Colitis, Gingival bleeding, Diarrhoea, Abdominal discomfort, Stomatitis, Nausea, Abdominal pain, Gastrointestinal haemorrhage, Abdominal distension, Abdominal pain upper, Dyspepsia, Constipation, Dysphagia, Mouth ulceration, Melaena, Vomiting	16
General disorders and administration site conditions	Pain, Drug ineffective, Multiple organ dysfunction syndrome, Chest pain, Disease progression, Chills, General physical health deterioration, Fatigue, Oedema peripheral, Mucosal inflammation, Pyrexia, Death, Malaise, Therapy non-responder, Treatment failure, Asthenia, Condition aggravated	17
Hepatobiliary disorders	Hepatic function abnormal, Hyperbilirubinaemia	2
Immune system disorders	Hypersensitivity, Graft versus host disease	2
Infections and infestations	Bacteraemia, Urinary tract infection, Bacterial infection, Cellulitis, Sepsis, Bronchopulmonary aspergillosis, Aspergillus infection, Fungal infection, Upper respiratory tract infection, Pneumonia, Infection, Neutropenic sepsis, Septic shock, Staphylococcal infection, Pneumonia fungal, Diverticulitis	16
Injury, poisoning and procedural complications	Product use in unapproved indication, Subdural haematoma, Contusion, Off label use, Toxicity to various agents, Product use issue, Fall	7
Investigations	Blood bilirubin increased, Full blood count abnormal, Aspartate aminotransferase increased, White blood cell count increased, Alanine aminotransferase increased, Platelet count decreased, Weight decreased, Red blood cell count decreased, C-reactive protein increased, Neutrophil count decreased, White blood cell count decreased, Blood creatinine increased, Haemoglobin decreased	13
Metabolism and nutrition disorders	Hyponatraemia, Hyperkalaemia, Hyperglycaemia, Dehydration, Decreased appetite, Tumour lysis syndrome, Hypokalaemia	7
Musculoskeletal and connective tissue disorders	Muscle spasms, Arthralgia, Pain in extremity, Muscular weakness, Musculoskeletal pain, Back pain, Bone pain, Myalgia	8
Neoplasms benign, malignant and unspecified (incl cysts and polyps)	Leukaemia recurrent, Myelodysplastic syndrome, Acute myeloid leukaemia recurrent, Acute myeloid leukaemia, Leukaemia, Malignant neoplasm progression, Neoplasm progression	7
Nervous system disorders	Cerebrovascular accident, Haemorrhage intracranial, Cerebral haemorrhage, Seizure, Somnolence, Headache, Syncope, Neuropathy peripheral, Dizziness	10
Pregnancy, puerperium and perinatal conditions		
Product issues		
Psychiatric disorders	Insomnia, Mental status changes, Anxiety, Confusional state	4
Renal and urinary disorders	Dysuria, Haematuria, Acute kidney injury, Renal impairment, Renal failure, Urinary retention	6
Reproductive system and breast disorders		
Respiratory, thoracic and mediastinal disorders	Cough, Interstitial lung disease, Pneumonitis, Lung infiltration, Acute respiratory distress syndrome, Respiratory distress, Pulmonary oedema, Oropharyngeal pain, Pleural effusion, Respiratory failure, Dyspnoea, Hypoxia, Organising pneumonia, Acute respiratory failure, Pulmonary embolism, Epistaxis, Haemoptysis	17
Skin and subcutaneous tissue disorders	Rash maculo-papular, Alopecia, Petechiae, Ecchymosis, Erythema, Rash, Urticaria, Acute febrile neutrophilic dermatosis, Pruritus, Hyperhidrosis	10
Social circumstances		
Surgical and medical procedures	Hospitalisation	1
Vascular disorders	Hypotension, Phlebitis, Haematoma, Deep vein thrombosis, Haemorrhage, Hypertension	6

When comparing the top 27 adverse reactions reported for each DNA methylation inhibitor in the SOC, all PTs are different for the inhibitors. Azacitidine and Decitabine both have unique symptoms in the areas of Blood and Lymphatic System Disorders, Cardiac Disorders, Gastrointestinal Disorders, General Disorders and Administration Site Conditions, Immune System Disorders, Infections and Infestations, Injury, Poisoning and Procedural Complications, Investigations, Benign, Malignant and Unspecified Tumors (including cysts and polyps), Nervous System Disorders, Psychiatric Disorders, Renal and Urinary Disorders, and Respiratory, Thoracic and Mediastinal disorders (Table 6).

**TABLE 6:** Different ADRs among two DNA methylation inhibitors.

System organ classes	Azacitidine	Decitabine
Blood and lymphatic system disorders	White blood cell disorder, Platelet disorder, Febrile bone marrow aplasia, Disseminated intravascular coagulation	Bicytopenia
Cardiac disorders	Atrial flutter, Cardiac failure acute, Angina pectoris, Cardiac disorder, Pericarditis, Cardiovascular disorder	Palpitations, Left ventricular dysfunction, Cardiomyopathy
Congenital, familial and genetic disorders		
Ear and labyrinth disorders		
Endocrine disorders		
Eye disorders		Vision blurred
Gastrointestinal disorders	Ascites, Haematochezia, Upper gastrointestinal haemorrhage, Gastrointestinal disorder	Proctalgia, Haemorrhoids, Ileus, Dry mouth
General disorders and administration site conditions	Injection site pain, Injection site reaction, Injection site erythema	Oedema, Drug ineffective for unapproved indication, Chest discomfort
Hepatobiliary disorders	Hepatic failure, Liver disorder, Cholecystitis	
Immune system disorders	Acute graft versus host disease	Acute graft versus host disease in intestine, Acute graft versus host disease in skin, Chronic graft versus host disease
Infections and infestations	Bronchitis, COVID-19, Sinusitis, *Clostridium difficile* colitis	Device related infection, Nasopharyngitis, Enterococcal infection, *Candida* infection
Injury, poisoning and procedural complications	Intentional product use issue	Product storage error
Investigations	Neutrophil count abnormal, Blast cell count increased, Transaminases increased, Full blood count decreased, Blood lactate dehydrogenase increased, Platelet count abnormal, Haemoglobin abnormal	Blood culture positive, Liver function test abnormal, Blood alkaline phosphatase increased, Lymphocyte count decreased, Hepatic enzyme increased, Haematocrit decreased, Body temperature increased
Metabolism and nutrition disorders		Hypophosphataemia, Failure to thrive, Diabetic ketoacidosis, Hypophagia, Cachexia, Hypoalbuminaemia, Hypervolaemia, Hypocalcaemia, Acidosis
Musculoskeletal and connective tissue disorders		Joint swelling
Neoplasms benign, malignant and unspecified (incl cysts and polyps)	Transformation to acute myeloid leukaemia, Myelodysplastic syndrome transformation, Differentiation syndrome, Chronic myelomonocytic leukaemia, Myelofibrosis	Acute myeloid leukaemia refractory
Nervous system disorders	Loss of consciousness	Lethargy, Tremor, Encephalopathy, Paraesthesia, Hypoaesthesia, Posterior reversible encephalopathy syndrome
Pregnancy, puerperium and perinatal conditions		
Product issues		
Psychiatric disorders	Depression	Delirium
Renal and urinary disorders	Renal disorder	Pollakiuria
Reproductive system and breast disorders		
Respiratory, thoracic and mediastinal disorders	Lung disorder, Respiratory disorder, Dyspnoea exertional	Rhinorrhoea, Pulmonary haemorrhage, Sputum increased
Skin and subcutaneous tissue disorders	Skin lesion, Skin reaction, Pyoderma gangrenosum, Rash erythematous, Rash pruritic, Rash macular	
Social circumstances	Blood product transfusion dependent	
Surgical and medical procedures		
Vascular disorders	Thrombosis	

## Discussion

Due to the inherent limitations of clinical trials, such as strict trial design, strict inclusion criteria, relatively small sample size, and limited follow-up time, the SRS has been used for safety assessment of suspected adverse events in drug vigilance. In addition, the research data from clinical trials may not conform to the real world where patients and comorbidities are heterogeneous. SRS plays an important role in signal identification (Lindquist et al., 2000). Currently, most research on drug safety signals mainly comes from three major databases: the EudraVigilance Data Analysis System (EVDAS), the Food and Drug Administration (FDA) Adverse Event Reporting System (FAERS), and the WHO-VigiBase^®^ (Vogel et al., 2020). WHO-VigiAccess, launched by WHO in 2015, aims to provide the public with information from VigiBase^®^, the global database of potential drug side effects reported by WHO. Data mining of the WHO-VigiAccess database will provide previously unknown drug AE associations and some established clinical associations (Yamoah et al., 2022).

This experiment aims to evaluate the post-marketing adverse events related to DNA methylation inhibitors in the WHO-VigiAccess database. The data from WHO-VigiAccess show that 38.38% of adverse events related to the two inhibitors come from Asia, followed by the United States. It is estimated that the incidence of MDS in the United States and Europe is 4.3 and 1.8 cases per 100,000 people per year, respectively. Some Asian countries report lower incidence rates, while estimates from other parts of the world are less frequent. In other research statistics, it can be found that the number of adverse events in the Americas and Europe is not much different from Asia, while the number of adverse events in Africa and Oceania is quite low. A large part of the reason comes from factors such as geographical and social environment, medical level, the lack of health professionals, the scarcity of medical knowledge, and the high costs caused by regional economic disparities, making Africa the region with the lowest adverse events (Ampadu et al., 2016).

In adverse reaction reports, males are more common than females, and the 65–74 age group has the most adverse reactions after treatment with methylation inhibitors, followed by a significant proportion in the ≥75 age group. Risk factors associated with MDS include older age and previous exposure to toxins, such as chemotherapy or radiotherapy. As age increases, physiological functions gradually decline, and the elderly often have various complications, affecting the metabolic process of drugs in the body and greatly increasing the risk of adverse events. Moreover, due to physiological differences between genders, the number of adverse events in males is more than 1.5 times that of females. Although adverse events occur in all age groups, the highest incidence rate is in the 65–74 age group (Itzykson et al., 2015).

AEs with a reporting rate >1% are usually considered the most common (Chen et al., 2019). Serious adverse events of the two DNA methylation inhibitors, including life-threatening events and hospitalization events, are not common, but the death event of azacitidine is 4.04%, which is much higher than decitabine. The most common adverse reactions of the two DNA methylation inhibitors are blood and lymphatic system disorders.

In a decitabine trial, 91% of patients reported grade 3 or four neutropenia, while 85% of patients reported grade 3 or four thrombocytopenia. In the decitabine registration trial, 87% of patients treated with decitabine experienced grade 3 or four neutropenia, while 50% of patients receiving supportive therapy experienced grade 3 or four thrombocytopenia. Furthermore, 85% of patients treated with decitabine experienced grade 3 or four thrombocytopenia, whereas the proportion of patients receiving only supportive therapy who experienced grade 3 or four thrombocytopenia was 43% (Fenaux et al., 2009; Kantarjian et al., 2006). There is no consensus on the best approach to managing myelotoxicity induced by hypomethylating agents. Potential strategies include dose delay, dose reduction, administration of hematopoietic growth factors, or simply waiting it out. This is related to the mechanism of action of azacitidine and decitabine in the body: after cellular uptake, azacitidine and decitabine are converted into their monophosphates, diphosphates, and triphosphates. Triphosphate decitabine is a deoxyribonucleotide that is incorporated only into DNA. Azacitidine is primarily converted into triphosphate azacitidine, which is incorporated into RNA. A small portion of the administered azacitidine (about 10%–20%) is converted by ribonucleotide reductase into 5-azacitidine triphosphate, which can be incorporated into DNA. Incorporation into DNA leads to the formation of adducts between DNA and DNMT-1. At high doses, DNA cannot be repaired and cell death occurs (Christman, 2002).

By December 2018, the FAERS database showed that the most common adverse reactions (≥30%) associated with venetoclax in combination with azacitidine or decitabine or low-dose cytarabine were nausea, diarrhea, thrombocytopenia, constipation, neutropenia, febrile neutropenia, fatigue, vomiting, peripheral edema, pneumonia, dyspnea, hemorrhage, anemia, rash, abdominal pain, sepsis, back pain, myalgia, dizziness, cough, oropharyngeal pain, fever, and hypotension. Through the VigiAccess database, we found that the top five adverse reactions related to azacitidine are febrile neutropenia (10.18%), neutropenia (6.67%), bone marrow suppression (6.14%), thrombocytopenia (6.03%), and anemia (4.65%). The top five adverse reactions for decitabine are bone marrow suppression (11.66%), neutropenia (9.3%), febrile neutropenia (8.12%), thrombocytopenia (7.66%), and leukopenia (5.24%). VigiAccess and FAERS, as databases for assessing post-marketing drug vigilance, show differences in the types and incidence rates of infection-related adverse reactions caused by two DNA methylation inhibitors. Since adverse events are voluntarily reported, passive monitoring of the FAERS database and the WHO-VigiAccess database cannot represent complete and comprehensive statistics. As a database for assessing post-marketing drug vigilance, WHO-VigiAccess shows that the types and incidence rates of infection-related adverse reactions caused by DNA methylation inhibitors vary. Since adverse events are voluntarily reported, the WHO-VigiAccess database cannot represent a complete and comprehensive statistical adverse event, and may lack information on reported events. This may require WHO-VigiAccess to provide more report information to the public to filter potential connections between drugs and adverse reactions to avoid incorrect guidance.

Another significant adverse event of DNA methylation inhibitor treatment is the greatly increased risk of infection (Quinto et al., 2014). Infection is a common and potentially fatal event affecting patients with myelodysplastic syndrome (MDS). A retrospective study showed that neutropenia and/or neutrophil dysfunction during the treatment of MDS patients; B cell, T cell, and NK cell defects; treatment toxicity; previous severe infections, and other factors can all lead to death (Merkel et al., 2013). Compared with the general population, the incidence and severity of infectious diseases in MDS patients are much higher. The increased risk of infection in these patients seems to be mainly attributed to immunosuppressive therapy. When DNA methylation inhibitors are used in combination for treatment, the risk of infection will also increase significantly (Derissen et al., 2013). And routine antibiotics, antifungal prevention does not seem to reduce the incidence of infection events. Considering the bacterial and fungal resistance risks associated with long-term use of anti-infective drugs, these drugs should be used cautiously for selected subgroups of MDS patients (Quinto et al., 2014). Clinical physicians exhibit significant variation in the use of antimicrobial prophylaxis during hypomethylating agent therapy. Some clinicians do not provide prophylactic measures and anticipate treatment of infections; others utilize antimicrobial prophylaxis, antifungal prophylaxis, antiviral prophylaxis, or some combination of these three. Currently, there are no randomized trials comparing various potential antimicrobial prophylactic strategies for MDS patients treated with azacitidine or decitabine, hence the basis for decision-making data comes from other settings.

Furthermore, MDS exhibits heterogeneity, not only due to diverse pathogenic mechanisms and morphological presentations but also in the natural course and outcomes of patients. The course of individual patients varies greatly, ranging from severely symptomatic diseases with a survival period limited to a few months to mildly symptomatic diseases with a survival period of 10 years or longer. Most MDS patients die from complications associated with severe cytopenias, rather than from the progression of leukemia.

If a hypomethylating agent is ineffective for a patient clinically, what reason is there to try another? Although azacitidine (5-azacitidine) and decitabine (5-aza-20-deoxycytidine) are chemically similar, differing only in a hydroxyl group on the sugar part, patients may respond to one compound and not the other for biological reasons. The cellular metabolism of azacitidine and decitabine is similar but not identical. After entering the cell through equilibrative nucleoside transporters (ENTs) on the cell surface, azacitidine is phosphorylated to 5-azacitidine monophosphate by uridine-cytidine kinase, while decitabine is phosphorylated by deoxycytidine kinase (the rate-limiting step for drug activation within the cell) (Kaminskas et al., 2005). In tumor cell lines, low expression of deoxycytidine kinase is associated with decitabine resistance but does not affect the metabolism of azacitidine (Qin et al., 2009). In cell lines, the correlation between sensitivity to decitabine and sensitivity to azacitidine is better than the correlation between sensitivity to azacitidine (Qin et al., 2009). Additionally, the overall hypomethylation pattern induced by azacitidine *in vitro* is different from that induced by decitabine (Flotho et al., 2009). These data suggest that some patients may be predestined to respond better to one hypomethylating agent than another, and 1 day it may be possible to obtain gene expression profiles before treatment to select the most appropriate drug.

The use of the spontaneous reporting system database has some important hidden limitations because reports are affected by notoriety bias, selection bias, and under-reporting (Quinto et al., 2014). As observed in the current study results that reported some AEs, the missing data cannot be attributed to either males or females, nor to age groups. In addition, because the World Health Organization’s VigiAccess database is cumulative data, the annual ADR cannot be obtained. When drugs are marketed at different times, the number of ADRs collected varies greatly, and it is impossible to compare the signal differences of all target inhibitors at the same time. Therefore, further data mining will not be possible. This study collected the number of ADRs and PTs over the years, compared the ADR.

The use of the spontaneous reporting system database has some significant implicit limitations, as reports can be influenced by notoriety bias, selection bias, and under-reporting (Kantarjian et al., 2006). As observed in the current study’s results, which reported some adverse events (AEs), the missing data cannot be attributed to males, females, or age groups. Moreover, because the World Health Organization’s VigiAccess database contains cumulative data, it is impossible to obtain the annual number of adverse drug reactions (ADRs). When drugs are marketed at different times, the number of ADRs collected varies greatly, making it impossible to compare the signal differences of all target inhibitors simultaneously. Therefore, further data mining cannot be achieved. This study collected the number of ADRs and preferred terms (PTs) over the years, comparing the ADR reporting rates of different drugs to avoid the impact of the timing of drug marketing.

## Data Availability

The original contributions presented in the study are included in the article/Supplementary Material, further inquiries can be directed to the corresponding authors.
