# Supplementary material for: DNA methylation inhibitors adverse reaction characteristic analysis: a descriptive analysis from WHO-VigiAccess
Source: Front Pharmacol. 2024 Oct 2;15:1470148. doi: 10.3389/fphar.2024.1470148 (PMC11479969; doi:10.3389/fphar.2024.1470148)
Supplement: Supplementary file 1 [file Table1.DOCX]

Supplementary Table1

| **System organ classes** | **Azacitidine** | | **Decitabine** | |
| --- | --- | --- | --- | --- |
|  | **RoR** | **PRR** | **RoR** | **PRR** |
| Blood and lymphatic system disorders | 0.86 | 0.88 | 1.17 | 1.13 |
| Cardiac disorders | 1.18 | 1.18 | 0.84 | 0.85 |
| Congenital, familial and genetic disorders | 1.05 | 1.05 | 0.95 | 0.95 |
| Ear and labyrinth disorders | 0.79 | 0.79 | 1.26 | 1.26 |
| Endocrine disorders | 0.68 | 0.68 | 1.47 | 1.47 |
| Eye disorders | 0.67 | 0.67 | 1.49 | 1.49 |
| Gastrointestinal disorders | 0.89 | 0.90 | 1.13 | 1.12 |
| General disorders and administration site conditions | 1.19 | 1.16 | 0.84 | 0.86 |
| Hepatobiliary disorders | 1.16 | 1.16 | 0.86 | 0.86 |
| Immune system disorders | 1.20 | 1.20 | 0.83 | 0.84 |
| Infections and infestations | 1.15 | 1.13 | 0.87 | 0.88 |
| Injury, poisoning and procedural complications | 0.74 | 0.75 | 1.35 | 1.34 |
| Investigations | 1.14 | 1.12 | 0.88 | 0.89 |
| Metabolism and nutrition disorders | 0.61 | 0.62 | 1.63 | 1.60 |
| Musculoskeletal and connective tissue disorders | 0.68 | 0.69 | 1.46 | 1.45 |
| Neoplasms benign, malignant and unspecified (incl cysts and polyps) | 1.61 | 1.58 | 0.62 | 0.63 |
| Nervous system disorders | 0.89 | 0.89 | 1.13 | 1.12 |
| Pregnancy, puerperium and perinatal conditions | 1.36 | 1.36 | 0.74 | 0.74 |
| Product issues | 9.42 | 9.40 | 0.11 | 0.11 |
| Psychiatric disorders | 0.79 | 0.79 | 1.27 | 1.27 |
| Renal and urinary disorders | 1.05 | 1.05 | 0.95 | 0.95 |
| Reproductive system and breast disorders | 0.89 | 0.89 | 1.13 | 1.13 |
| Respiratory, thoracic and mediastinal disorders | 0.84 | 0.85 | 1.19 | 1.18 |
| Skin and subcutaneous tissue disorders | 1.36 | 1.34 | 0.74 | 0.74 |
| Social circumstances | 1.68 | 1.68 | 0.60 | 0.60 |
| Surgical and medical procedures | 0.98 | 0.98 | 1.02 | 1.02 |
| Vascular disorders | 1.05 | 1.05 | 0.95 | 0.95 |
